# Empowering Students in Transition

**DOI:** 10.3389/fpubh.2016.00211

**Published:** 2016-09-28

**Authors:** Ann-Catherine Sullivan

**Affiliations:** ^1^Department of Health and Sport Sciences, Otterbein University, Westerville, OH, USA

**Keywords:** transition program, fitness, peer modeling, collaborative learning, empowerment theory

## Abstract

The purpose of this study was to (a) identify potential benefits for students with disabilities taking part in a physical activity program with same-age typical peers on a Midwest university campus and (b) to determine if the program impacted the students with disabilities empowerment. Empowerment theory was used to determine how transition students’ attitudes change over the course of the semester while participating in a workout buddy program with same-age college peers. The program was structured to provide a sense of empowerment to students to make their own decisions and learn for themselves so they do not feel a lack of power in their lives. This study implemented elements of a quantitative design but a majority utilized a qualitative design based on the assumptions of the Interpretivist paradigm. The quantitative design elements focused on the analysis of two questionnaires: Sports Questionnaire and the Perceived Control Scale Questionnaire. The analysis of the focus group data revealed the following themes as positive effects of the intervention: positive effect on empowerment, how happy the program made the students, what benefits the students gained from the program, the student’s familiarity with university students, and the environment, and, lastly, the students ability to ask for assistance when need. Findings from the study determined that the empowerment of the students with disabilities was impacted while participating in the program. In general, the findings of gaining empowerment were similar to previous studies in that students with disabilities are able to gain empowerment from participation in fitness and recreation programs. The researcher noted during focus groups that some of the Best of Both Worlds (BOBW) students were not confident in starting conversations with their university peers. Although the BOBW students felt a sense of losing empowerment with this specific instance, there was an overall positive impact on the BOBW students’ empowerment. By giving the students the opportunity to participate and socialize with peers of their own age at a college setting, they were able to gain a sense of empowerment in their own life.

## Introduction

The Passage of Public Law 94–142, and all subsequent reauthorizations of the Individuals with Disabilities Education Act (IDEA), has resulted in increased opportunities for individuals with disabilities in schools and recreation settings (1). Prior to this law, students with identified disabilities were often denied an education, cognitive age was used to deny instruction, testing was discriminatory and an emphasis was placed on the disability label versus the needs of the individual (2). The Americans with Disabilities Act has lead to increased opportunities for individuals with disabilities in schools and recreation settings (1).

The Best of Both Worlds (BOBWs) Program is a comprehensive collaborative transition program for students, aged 19–21, who have identified disabilities. The program provides instruction in work skills, community participation skills, independent living skills, and self-advocacy skills. BOBW students have completed credits required for high school graduation in the state and are accessing post-secondary training alongside their typical same-aged peers on a university campus. BOBW’s students were selected for program participation by their Individualized Education Program (IEP) team, based on additional skills needed to live, work, and advocate for themselves as adults in the community. The program has been in place since the beginning of fall semester, 2011, focusing on socialization, gaining independence as well as a fitness/recreation component. Within the program, there are 1.5 intervention specialists and 4 job coaches on site. BOBW students are accompanied by job coaches, during all work, community, and fitness/recreation activities, to help answer any questions that arise while they are offsite. This support makes for an easier transition toward independence for the students.

The BOBW program has a very rich and multifaceted partnership with a small Midwest university whereby same-age college peers are partnered with and work alongside the BOBW students as they develop a healthy, active adult life, through a peer buddy workout program, community service, and social opportunities. The program provides the BOBW students with encouragement to become more independent by offering safe recreation and leisure skills, teaches safe equipment utilization, and a workout facility and opportunity to exercise with same-aged peers. The university students, who serve as peer buddies, also model daily social skills that are acceptable to society, as well as health factors related to using a workout facility for recreation and leisure in adult life (e.g, using a swipe card to access the facility, wiping down machines after usage, etc.). Additionally, a Certified Adapted Physical Educator (C.A.P.E.) professor works within the program, matching buddies, offering feedback for program enhancement, and teaching the “buddies” how to teach self-advocacy skills to the BOBW students. Self-advocacy is one of the largest curriculum areas in the transition program life skills curriculum. Students with disabilities need training to understand self-advocacy, how it applies to them, and how to use it in their daily lives.

The purpose of this study was to explore how participation in a cardiovascular exercise, weight training, and recreation program impacted the empowerment of students with disabilities when they participated in the program with an assigned university “buddy.” More specifically, the research set out to determine how the participants’ attitudes and empowerment changed while participating in BOBWs Workout Buddy Program. Studies have shown that there is a positive correlation between physical activity and empowerment for students with disabilities (3, 4). In a past study, the biggest barrier found was that fitness and recreation facilities were unfriendly environments (5). There is limited knowledge about empowerment theory in a college campus fitness program and with a transition program. This research intended to determine how the participants’ attitudes and empowerment changed while participating in BOBWs Workout Buddy Program, as additional research was needed in this area. This research is significant because it addresses the importance of empowerment in students with disabilities. An important supplemental purpose was to identify and break down the barriers those adults with disabilities face with daily participation in fitness and recreation.

Empowerment Theory focuses on the process of increasing personal power, meaning self-confidence, and self-determination, in order to enable individuals, families, and communities to improve their situation (3, 6). According to Depauw and Doll-Tepper (7), historically, teachers made uninformed choices about children with special educational needs that in effect disempowered them. By not allowing students to make their own choices and decisions teachers ignore the importance of children’s lived experiences of their own difficulties. The teachers’ limited understanding of the child’s needs, essentially restricted successful inclusion (7). Adults with disabilities typically feel a lack of power in their lives, which could impede their sense of making their own decisions or necessary changes in their own lives (3, 6, 8). Empowerment theory takes the environment into account, as well as promotes positive behaviors and participation that involve individuals to improve their overall quality of life (9).

By allowing children with special educational needs to take on challenges, and encourage them to participate, children will succeed. As such, the consultation and empowerment of children with special educational needs with regard to their education is of great importance. It allows the child a chance to accept and cope with their disability, as well as providing fundamental information to adults regarding the child’s experiences (10) (p. 174).

This may help them later on in life so they can make their own decisions, which is necessary in life (3, 11). However, previous research found that BOBW buddies sometimes felt uncomfortable being around adults with disabilities (12). Similarly, Griffin et al. (13) noted “concerns for students that were not as willing to participate with students with disabilities included that they might not know how to act around the students with disabilities, which in turn would make them feel uncomfortable” (13) (p. 235).

While participating with a fitness program, adults with disabilities start to learn how to work their way around the restrictions they face (5). Providing adults with disabilities the opportunity of participation and involvement, BOBW program provides them a sense of empowerment to make their own decisions and learn to become independent. Through participation with fitness and recreation programs, adults with disabilities learn to overcome self-imposed perceptions of their capabilities as well as how to turn their limitations into abilities (14). People who perceive themselves as competent, capable, and self-determining will be able to face and deal with life’s challenges (9).

According to the Centers for Disease Control and Prevention, adults need at least 150 min of moderate-intensity aerobic activity every week and muscle-strengthening activities on two or more days a week (15). Exercise has been shown to have a strong correlation between participation in an exercise program and positive changes in behavior for adults with disabilities (16–18). Findings from studies suggest that participation in exercise programs can alter behaviors, such as intellectual functioning, stereotypic behavior, work performance, and self-concept (16–18). Gabler-Halle et al. (16) suggested that students with disabilities should be provided with encouragement to choose activities that they find enjoyable. This helps in their comfort level toward being at the gym with others around them. The students should carry out activities voluntarily so they are enjoying themselves rather than being forced to participate (16).

Adults with disabilities may feel a lack of power in their lives, which later on in life could impede their sense of making their own decisions or necessary changes. While participating in a fitness program, adults with disabilities start to learn how to work their way around the restrictions they face. Block et al. (9) found that providing adults with disabilities the opportunity to participate, they were provided a sense of empowerment to make their own decisions and learn to become independent. Coates and Vickerman (14) found that students with disabilities felt that physical activity helped them relieve stress and made them feel happiness, and gave them mostly positive feedback. However, negative feedback was directly related to negative attitudes from their peers or surroundings (14).

## Barriers to Participation in Fitness and Recreation

There are many barriers that adults with disabilities face with daily participation in fitness and recreation. These barriers consist of health, social, and familiarity with environment barriers. The BOBW Program has been constructed with the intention of eliminating these barriers.

### Familiarity with Environment Barriers

Best of Both Worlds students in particular faced the barrier of coming to a campus full of students with whom they are unfamiliar. They also faced a barrier of working out in an environment in which they were originally unfamiliar. The university students were able to observe the BOBW students utilize the campus as a *typical college student*, as well as how much this meant to the BOBW students. With the university students *approval*, BOBW students were provided the empowerment to become an individual and feel comfortable with coming to campus and being around other students.

During their workout, there were many college students working out as well, which could have been intimidating at times for the BOBW students. With the BOBW program, buddies eased the students into the workouts and tried to increase socialization with other college students as well. The greatest emotional and psychological barriers adults with disabilities face when wanting to use fitness and recreation facilities were unfriendly environments (5, 13).

### Social Barriers

Individuals with disabilities often witness negative attitudes and behavior from students without disabilities or the facility staff (13). Other concerns include not having anyone to assist individuals with disabilities, when needed, and lack of support from friends and family to access and participate in fitness programs. Outsiders negative attitudes toward individuals with disabilities could potentially make the students feel uncomfortable and unwilling to come back (19). These negative attitudes can have a huge effect on the students and could potentially make the students feel uncomfortable and unwilling to come back (19).

Adults with disabilities may feel a lack of power in their lives, which later on in life could impede their sense of making their own decisions or necessary changes. While participating in a fitness program, adults with disabilities start to learn how to work their way around the restrictions they face. Through participation with fitness and recreation programs, adults with disabilities learn to overcome self-imposed perceptions of their capabilities as well as how to turn their limitations into abilities. People who perceive themselves as competent, capable, and self-determining will be able to face and deal with life’s challenges (9).

Griffin et al. (13) found that college students indicated positive attitudes toward students with disabilities but they also expressed concerns that they might not know how to act around the students with disabilities, which in turn would make the students with disabilities feel uncomfortable. This study also showed that females were more willing to participate and interact with students with disabilities than were males (13). Previous BOBW program research noted that freshman buddies entering the program felt reluctant to work with students with disabilities due to minimal previous experience (12). However, as the buddies attended more sessions with the students, they began to feel more comfortable, which made the environment more welcoming. Once the buddies eliminated the barrier of being uncomfortable, they were able to begin building a relationship with the students. In the long run, it helped the students because they began to feel more comfortable speaking up for themselves. Students with disabilities have been found to be more willing to listen to someone their own age rather than their job coach who was much older (19). They also liked to have age appropriate conversations and, therefore, providing buddies who are around the same age gives them the opportunity.

### Health Barriers

There are many health issues that our society faces today. Such issues include: disability; obesity; access to facilities; and equipment, etc. Students with disabilities tend to have lower fitness levels due to the lack of participation in physical activities; therefore, progressions and modifications to physical activities are needed (20). Obesity is a serious problem that citizens in the United States face on a daily basis. Green and Reese (21) found that providing individuals with disabilities the opportunity to become empowered and be able to feel comfortable around others at the gym provides them with opportunities to obtain a healthy lifestyle (21).

### Barriers Accompanied by Supports

Having committed buddies to help guide and encourage students to participation in physical activity makes for a better environment. Block et al. (9) found that trying to eliminate barriers associated with disabilities and physical exercise help motivate and increase physical exercise in the students. Research has shown that if the buddies are around the same age as the students with disabilities, the program may be more successful to help keep the student engaged and motivated (9) Previous BOBW program research noted that the BOBW students like to have age appropriate conversations and providing buddies that are around the same age provides that opportunity (12).

## Methods

This study implemented a mixed method design based on the assumptions of the Interpretivist paradigm. This study involved the students of BOBWs students and university buddies over a 3-month period. There was a constant setting at the recreation facility on the university campus. Two questionnaires were implemented: Sports Questionnaire (14) and the Perceived Control Scale Questionnaire (22). The Sports Questionnaire and the Perceived Control Scale questionnaire were used once toward the start of the program and once toward the end. Targeted questions from the initial implementation of each of the surveys added to the demographic data for the BOBW students. The scores of the questionnaires were calculated by questionnaire category and the data were illustrated in a pie chart or box plot as applicable.

### Subject Selection and Gaining Access to the Site

The researcher has worked with the program since its inception in 2011. The BOBW student participants were selected due to the researcher’s previous experience, which led her to contemplate if participation in the BOBW workout Buddy program impacted the empowerment of the BOBW students. The fall 2015 was an ideal year for implementation of this study because 10 new students were starting the BOBW program. Students and buddies attended the typical routine of working out for an hour session, twice a week in the university campus fitness center. University students served as workout buddies and mentors to motivate, interact, and exercise weekly with students enrolled in the BOBWs Program. The BOBW program students had individualized workout programs created by the public school adapted physical educator, which were designed to include the Cybex and cardiovascular machines available in the fitness center on the university campus. The BOBW students and buddies serving as college buddies worked out for 1–1.5 hours twice weekly. Other opportunities were available to socialize on campus, such as use of recreation facilities, athletic contests, lunch, theater productions, and other campus programs with their workout buddies.

### Participant Demographics

As illustrated in Table 1, the participants were the students enrolled in the BOBW Program who submitted a signed consent form. Although there were 17 students enrolled in the program, only 8 students completed the Consent for Participation in Social and Behavioral Research form and participated in the study. As noted in Table 2, the participants consisted of three males and five females. The mean age of the participants was 19.5 years of age.

**Table 1:** **BOBW student population**.

Gender	Male	Female
All BOBW students (*N* = 17)	9	8
Students in study (*N* = 8)	3	5

**Table 2:** **BOBW student population by gender and assigned number**.

Assigned number	Male	Female	Year in program
Student 2		X	1st
Student 3	X		1st
Student 7	X		1st
Student 8		X	2nd
Student 12		X	1st
Student 13		X	1st
Student 14		X	2nd
Student 16	X		2nd

### Questionnaire Selection

For this research study, two questionnaires were used: the Perceived Leisure Control questionnaire (22) and the Sports questionnaire (14) that was subdivided into two questionnaires containing sports interests and participation section as well as an *About Me* section. The surveys were reviewed by a panel of experts comprised the following: two Adapted Physical Educators, one Adapted Physical Education Professor, and two public school Intervention Specialists who were familiar with the BOBW program and students. The expert panel reviewed the surveys to determine content validity, appropriateness for the population, and appropriateness of the scale. The expert panel determined that the surveys contained the appropriate content that was intended to be measured, and it was determined that they survey had content validity. The Expert Panel also evaluated the instruments relative to presence of construct of interest and determined that construct was addressed in the instrument.

The Perceived Control Scale questionnaire was composed of 17 questions about the BOBW students and how they felt about participation in sports/activities. This questionnaire helped the researcher to better understand where the BOBW students stood on their opinion of their own empowerment. The answers were based on a five-point Likert scale as follows: 1-Stongly agree; 2-Agree; 3-neutrual; 4-Disagree; and 5-Strongly disagree. This questionnaire was used as a pre/post-questionnaire to determine any differences in empowerment.

The Sports Questionnaire (14) was used to acquire specific information about the types of physical activities in which the students participate. At the end of the Sports questionnaire, an “About Me” section was provided to get insight on how the students felt about their sport ability and how they felt around their peers while performing recreation activities. This questionnaire helped to better understand the types of activities with which the students would like to be involved. The first part of the survey consisted of five questions asking the students what sports they have participated in before and with which sports they would like to participate. Under each of the five questions, there were 20 sports/activities to place a mark next indicating that they are choosing this sport/activity. There was also a blank space where the BOBW students were able to enter another sport/activity that was not listed. The *About Me* section of the survey consisted of nine questions. These questions asked the BOBW students about their feelings toward the program and their own performance throughout the program. The answer options to the questions were as followed: Yes, with a smiley face; Not sure, with a straight face; No, with a frown face. This questionnaire was used as a pre/post-questionnaire to witness any differences in empowerment.

The Perceived Leisure Control and Sports questionnaires were administered twice to each participant, once at the beginning of the fall program and a second time toward the end of the fall program; before the focus groups were conducted. The purpose of the redelivery of each questionnaire was to determine if the BOBW students’ empowerment had changed from the beginning of the program to the end of the program. The surveys were taken by paper and pencil. An Intervention Specialist or trained job coach were in the same room to provide support to the BOBW students, in accordance with the student’s Individualized Education Plans.

#### Procedures

This study obtained approval from universities’ institutional Review Board (IRB) and was carried out in accordance with the recommendations of the board. Additionally, written consent to conduct the study was obtained from city school system and written consent for participation in the study from study participants or their legal guardians was obtained in advance in accordance with the Declaration of Helsinki. Informational letters were sent to the parents/guardians of the BOBWs students, who were not their own guardians to explain the study and surveys that their children would be completing. Participants (or their legal guardians) who were willing to participate in the study were asked to sign the consent form. If participants, or their legal guardians, did not want to participate in the surveys or focus groups, they were dismissed from the study without any penalty.

### Trustworthiness of Data

Trustworthiness was a strategy used in this study to assure credibility of the data. Four strategies were used throughout this research project: member checking, identifying negative cases, peer debriefing, and triangulation of data (23, 24). The researcher attempted to establish credibility by becoming familiar with the setting and participants prior to the start of the study. Peer debriefing sessions were completed with the researcher’s advisor. Data sources were triangulated and negative cases were sought.

Data triangulation was a method used to ensure consistency in data by collecting data through multiple methods. Patton (25) stated “studies that use only one method [of data collection] are more vulnerable to errors linked to that particular method … than are studies that use multiple methods in which different types of data provide cross-data validity checks” (25) (p. 1192). For this reason, four different types of data collection occurred in this study. Specifically, triangulation occurred in this study by collecting three different participants questionnaires and the focus group transcripts. Triangulation of sources is checking for consistency of data from the same source and methodology.

At each stage of data collection, peer debriefing and analysis of negative cases took place. Peer debriefing involved the researcher meeting with her advisor to discuss initial data findings and themes found in data review to ensure that similar conclusions were made about the data. The data were also assessed for negative cases. Negative case analysis was used to broaden an already established theory, display an exception to a theory or phenomenon, or completely change a theoretical framework (25). Negative cases also show how a specific group, environment, dynamic, or instance does not fit a pre-established pattern or theory of interaction already prevalent in literature. The data collected were assessed for negative cases to see the themes that emerged that were not previously described in the literature or did not follow common themes found in previous research (25).

### Focus Groups

Focus groups were used as a form of interviewing with the intention of providing another layer of data or perspective on the research problem (26). Focus groups were conducted to help determine whether or not student’s empowerment had changed throughout the BOBW program. Four separate focus groups were conducted based on the BOBW student’s availability. All focus groups were conducted in a classroom located in the BOBW program building. The focus groups were convenience scheduled in accordance with the researchers schedule and the BOBW student’s schedules.

## Questionnaire Results

The first result section pertains to the questionnaires. The second result section includes the focus group data to support the question of this study.

### Sports Questionnaire *About Me* Section

The data from implementation of the pre and post *About Me* section of the Sports questionnaire were illustrated in the pie charts for questions 1–9. Pie charts were used to show proportional data and the percentage represented by Yes, Maybe, and No responses to the *About Me* section of the Sports Questionnaire. A separate pie chart was created for each question within the *About Me* section of the Sports Questionnaire.

Question number one asked if the students enjoyed the BOBW program. Question number two asked if the buddies for the BOBW program helped enough during the workout. This pie chart compares the answers from pre- to post-questionnaires showing that they were answered the exact same way for both question one and two. All students reported that they enjoyed the program from the beginning of the semester to the end, making the BOBW program a positive aspect in their life.

**Figure 1:**
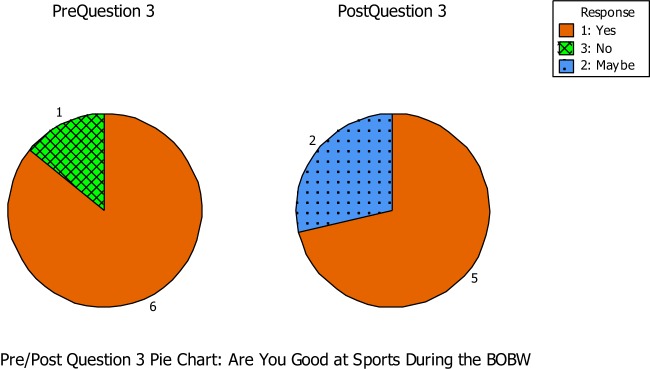
**Question number three asked if the students believe they were good at sports during the BOBW program**.

Question number three asked if the students believe they were good at sports during the BOBW program (see Figure 1). During the pre-questionnaire, six out of the seven students believed that they were good at sports during the BOBW program, but one student felt she was not. During the post-questionnaire, five out of the seven students believed they were still good at sports during the BOBW program, while two of the students were not really sure if they were or not. The researcher noted a small sense of empowerment on Student 13 who was a first year student in the BOBW program. She reported that she did not believe she was good at sports during the pre-questionnaire, but reported that she was not sure for the post-questionnaire. Student 12 showed a negative impact from the beginning of the semester to the end when asked if she believed she was good at sports. She reported that she was good at sports during the pre-questionnaire but was not sure during the post-questionnaire.

**Figure 2:**
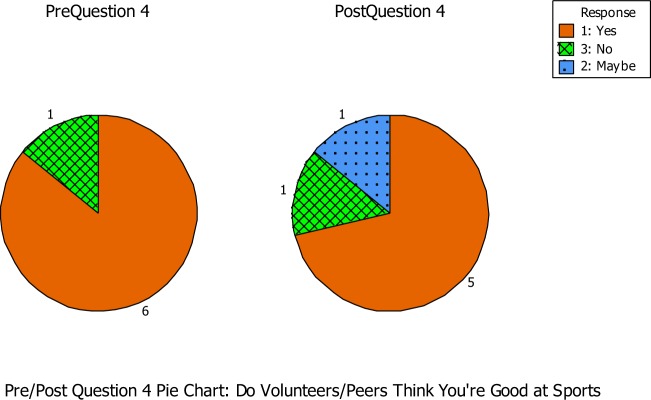
**Question number four asked if the students felt that the buddies/peers think that they were good at sports**.

Question number four asked if the students felt that the buddies/peers think that they were good at sports (See Figure 2). During the pre-questionnaire, six out of the seven students agreed that their buddies/peers thought they were good at sports, while one student did not. During the post-questionnaire, five out of seven students agreed that the buddies/peers thought that he/she was good at sports. However, one student was unsure and the other student disagreed that the buddies/peers thought she was good at sports. During the pre-questionnaire, Student 2 reported that she did not think her buddies/peers think she is good at sports. But during the post-questionnaire, she reported that she was unsure if her buddies/peers thought she was good at sports. This was a small instance of empowerment shown in the program. Student 13 had a negative impact from this question. During Student 13’s pre-questionnaire, she reported that her buddies/peers thought she was good at sports. However, in the post-questionnaire, she reported that her buddies/peers did not think she was good at sports. This was an instance of negative impact on empowerment for Student 13. She may have believed her buddies/peers thought she was good at sports until she actually played sports around them during the program. This could be the reason for her loss of empowerment.

**Figure 3:**
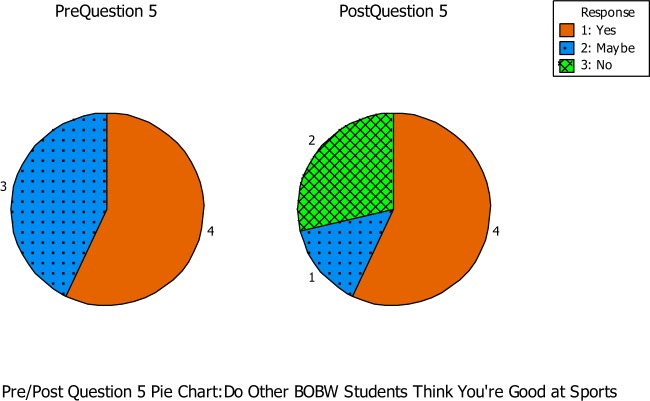
**Question number five asked if the students felt that other students in their class thought they were good at sports**.

Question number five asked if the students felt that other students in their class thought they were good at sports (see Figure 3). During the pre-questionnaire, four out of the seven students thought that their classmates thought they were good at sports, while the other three students were not sure. During the post-questionnaire, four out of the seven students indicated that their classmates thought they were good at sports, while two students did not and one other student was unsure. Student 16 had a small positive impact due to this question. He reported that he was unsure if others in his class thought he was good at sports during the pre-questionnaire. But during the post-questionnaire, he reported that he thought others in his class thought he was good at sports. He may have been unsure at the beginning of the program, but was reassured by his classmates throughout the semester. Students 2 and 13 seemed to have a negative impact on empowerment from pre- to post-questionnaire. This instance could be because these students had never been in a program, such as the BOBW, so they were not sure how other students in their class felt toward them until after the semester had ended.

**Figure 4:**
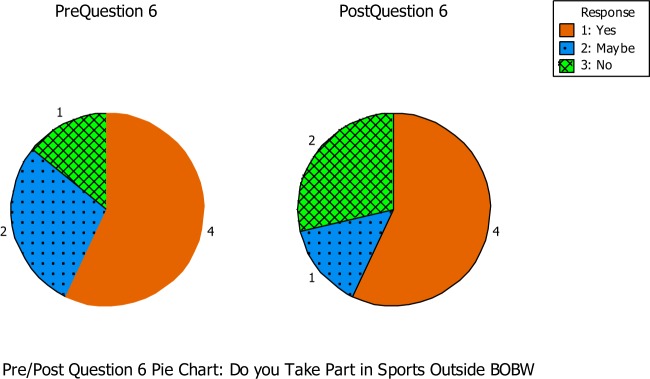
**Question number six asked if the students took part in any sport outside of the BOBW program**.

Question number six asked if the students took part in any sport outside of the BOBW program (See Figure 4). During the pre-questionnaire, four out of the seven students indicated that they took part in another sport, while two students were unsure and one student did not. During the post-questionnaire, four out of the seven students again answered yes, while one student was unsure and two students said they were not apart of a sport outside of the BOBW program.

**Figure 5:**
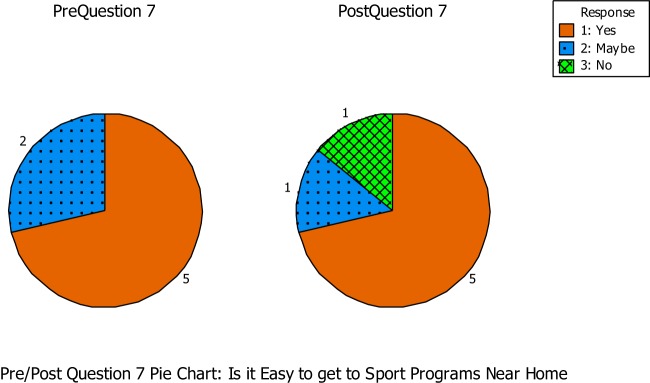
**Question number seven asked if the students thought it was easy to get to sport programs near where they live**.

Question seven asked if the students thought it was easy to get to sport programs near where they live (See Figure 5). During the pre-questionnaire, five out of the seven students answered yes, while the other two students were unsure. During the post-questionnaire, five out of the seven students answered yes once again, while one student answered unsure and the other answered no.

**Figure 6:**
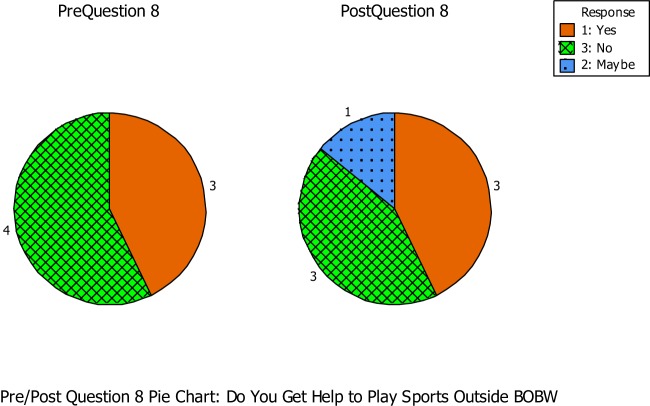
**Question number eight asked if the students got any help to play sports outside the BOBW program**.

Question number eight asked if the students got any help to play sports outside the BOBW program (See Figure 6). During the pre–questionnaire, three out of the seven students answered yes, while the remaining four students answered no. During the post-questionnaire, three out of the seven students answered yes, while one student was unsure and the remaining three students answered no.

**Figure 7:**
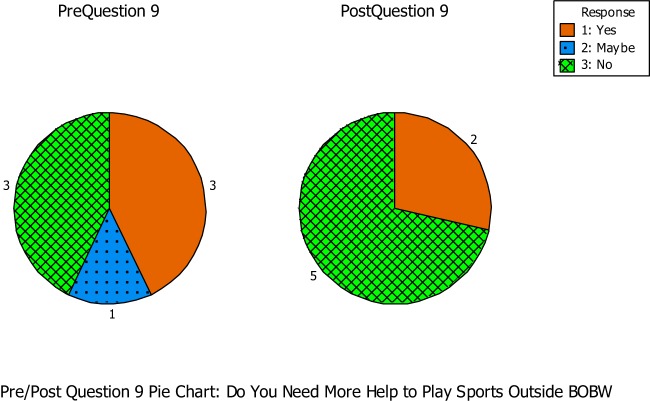
**Question number nine asked if the students thought they needed more help to play sport outside the BOBW program**.

Question number nine asked if the students thought they needed more help to play sport outside the BOBW program (see Figure 7). During the pre-questionnaire, three out of the seven students said yes, while one student was unsure and the remaining three students said no. During the post-questionnaire, two out of the seven students indicated that they needed help to play sports outside the BOBW program, while the remaining five students indicated no. Students 13 and 14 reported a positive impact on empowerment. During the pre-questionnaire, student 13 stated she was unsure if she needed help outside the BOBW program. But during the post-questionnaire, she stated she did not think she needed more help to play sports outside the BOBW program. Student 14 showed the same instance of positive impact on empowerment. However, Student 14 reported yes during the pre-questionnaire and no during the post-questionnaire. This instance showed a positive impact from the BOBW program to become more independent while at other sports and on the student’s lives.

### Perceived Leisure Control Questionnaire

The data from the Perceived Leisure Control Questionnaire were illustrated in Box plots with the intention of visually representing the data. Box plots are useful for identifying outliers as well as for comparing data distributions. The confidence level was set at 80% due to the small size of the population with the intention of illustrating the degree of dispersion of data, as well as the outliers between the pre- and post-implementation. The null hypothesis (H0) concluded that all pre- and post-questions were answered the same. The alternative hypothesis concluded that pre- and post-questions differ.

**Figure 8:**
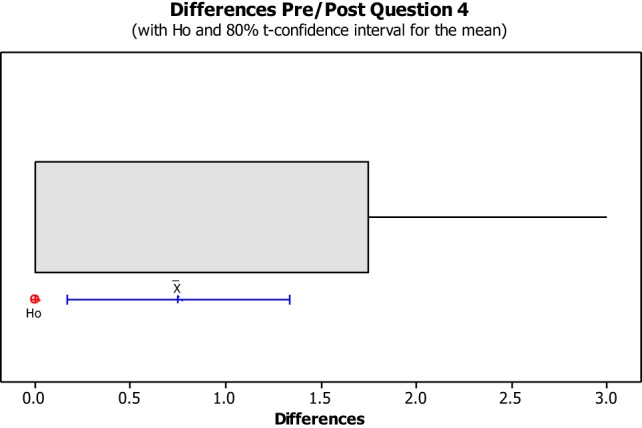
**Question 4 stated, “If someone started an argument with me, I could make him or her stop.”** This boxplot shows us the confidence interval (blue line margin), with the mean marked as an x on the boxplot at an 80% confidence level and the position of our null hypothesis value at 0.

**Figure 9:**
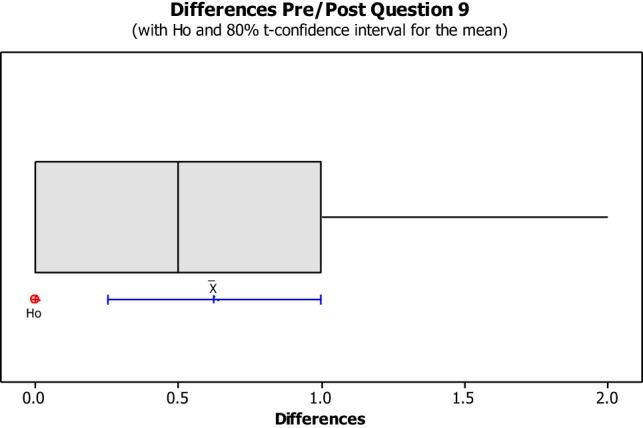
**Question 9 stated “I can make good things happen when I do recreation activities.”** This boxplot shows us the confidence interval (blue line margin), with the mean marked as an x on the boxplot at an 80% confidence level and the position of our null hypothesis value at 0.

**Figure 10:**
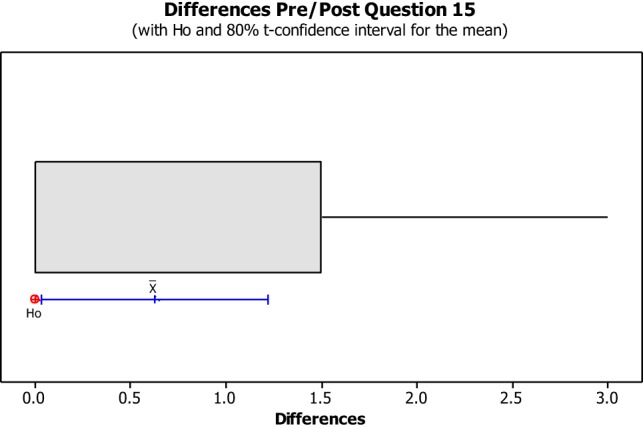
**Question 15 stated, “I can do things during recreation activities that will make other people like me more.”** This boxplot shows us the confidence interval (blue line margin), with the mean marked as an x on the boxplot at an 80% confidence level and the position of our null hypothesis value at 0.

No data dispersion was found pertaining to questions 1, 2, 3, 5, 6, 7, 8, 10, 11, 12, 13, and 14. The pre/post-questionnaire boxplots for questions 4, 9, 15, 16, and 17, created with an 80% confidence interval, the H0 was rejected as all answers for pre- and post-questionnaire questions were answered the same. As noted in Figures 8–10, the boxplot show the confidence interval with a blue line margin, with the mean marked as an x on the boxplot at an 80% confidence level and the position of the Ho value at 0. Since the Ho fell outside of the confidence interval on questions 4, 9, 15, 16, and 17; therefore, the hypothesis was rejected.

Question number four asked the students if someone started an argument with me, I could make him or her stop (See Figure 8). This question showed no impact on four out of the eight students, while the other four students had a negative impact from the beginning of the semester to the end of a semester. Student 8 showed an instance of negative impact and loss of empowerment from this question. This finding was interesting because the student is a second year in the program. Originally, she reported she strongly agreed that she could make someone stop if they started an argument with her. During the post-questionnaire, Student 8 reported that she disagreed with that statement.

Question number nine asked the students if he/she could make good things happen when he/she did recreation activities (See Figure 9). This question showed no impact on four out of the eight students, while the other four students seemed to have a negative impact. Question number 15 asked the students if he/she could do things during recreation activities that would make other people like them more (See Figure 10). This question showed no impact on six out of the eight students, while the other two students seemed to have a negative impact from the beginning of the semester to the end of the semester. Student 16 showed a negative impact from this question reporting that he agreed with the statement made in question fifteen. However, during the post questionnaire, he reported that he strongly disagreed with the statement made in question fifteen.

**Figure 11:**
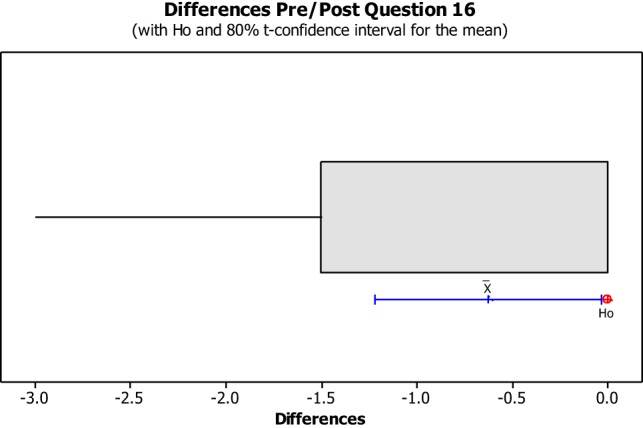
**Question 16 stated, “I can make recreation activities fun for everyone.”** This boxplot shows us the confidence interval (blue line margin), with the mean marked as an x on the boxplot at an 80% confidence level and the position of our null hypothesis value at 0.

Question number 16 asked the students if he/she could make recreation activities fun for everyone (See Figure 11). This question showed no impact on six out of the eight students, while the remaining two students seemed to have a positive impact. Two students seemed to have a positive impact on this question from pre-questionnaire to post-questionnaire. Both Student 12 and 16 strongly agreed with the statement made in question 16 during the post-questionnaire showing an instance of gaining empowerment from the BOBW program.

**Figure 12:**
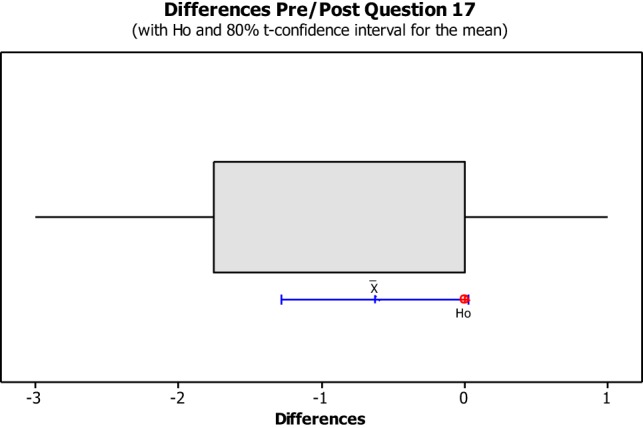
**Question 17 stated, “I can do things in my recreation activities that will help other people win more often.”** This boxplot shows us the confidence interval (blue line margin), with the mean marked as an x on the boxplot at an 80% confidence level and the position of our null hypothesis value at 0.

Question number 17 asked the students if he/she could do things in their recreations activities that would help other people win more often (See Figure 12). This question showed no impact on four out of the eight students, while one student had a negative impact and three students had a positive impact. Three students showed a positive impact due to this question. Both Students 13 and 16 strongly agreed with the statement made in question 17 during the post questionnaire showing an instance of gaining empowerment from the BOBW program.

## Focus Group Results

The *apriori* and notable themes emerging from the data were identified and constantly compared with previous data. Strauss and Corbin (27) suggested that after data collection the researcher should analyze the data using a constant comparative method. Triangulation methods included crosschecking sources of data to improve credibility (24). This process was utilized to confirm the researcher’s interpretations. The comparison of multiple data sources allowed the identification of inconsistent or unclear information to be identified and clarified.

The data collected through the participants’ questionnaires and transcribed focus group interview data were analyzed using QSR International NVIVO 10 for Windows qualitative software with open coding technique, which involved the researcher scanning the responses for themes or concepts found throughout the data. Once open coding was complete, axial coding was completed to formulate more encompassing categories of phenomenon based on the themes found with open coding. This was completed in the hope of finding connections between different themes found in the data. Finally, selective coding was completed in the hopes of developing an overall explanation for why the themes occurred from study.

### Focus Group Data Discussion

Based on the review of literature, there were five *a priori* themes sought during the focus group data analysis. These five themes consisted of positive effect on empowerment, how happy the program made the students, what benefits the students gained from the program, the student’s familiarity with the university peer buddies, and the environment, and, lastly, the students ability to ask for assistance when they need it. The analysis of the focus data revealed the following themes as positive effects of the intervention.

#### Positive Affect on Empowerment

There were a total of 32 references made pertaining to the affect on empowerment. Within this theme, there were three subthemes identified as follows: comfortable; confidence; and independence. The findings noted that the references made within this theme came from all four focus group data sources.

The participants made the following statements about becoming comfortable:

Yes, when I first came back from my surgery I was a little uncomfortable. I’m a very shy person; I’m very quiet and sometimes not talkative at all. I am talkative with my buddies now (Focus Group 1).

Like spending time with workout buddies and spending time with them on campus. Easy to get along with. Easy for me to introduce myself (Focus Group 2).

I am more outgoing because of buddies. When I meet new people, I get really nervous and when I am with my buddies, I feel more comfortable (Focus Group 2).

Pretty good, cool to hang out with; feel more comfortable (Focus Group 3).

Feel comfortable telling buddies if weights are too heavy, etc. Feel more comfortable with buddies now, as compared to beginning of school year (Focus Group 4).

One negative instance was found when a participant stated “I feel good, no intimidation. Sometimes I feel nervous around them (buddy) when someone approaches me to talk” (Focus Group 4).

The participants made the following statements about gaining confidence:

…more confidence with machines (Focus Group 2).

Feel a little more outgoing; feel more comfortable on campus (Focus Group 3).

Feel like I can talk to more peers (Focus Group 3).

…feel safe with buddy and willing to joke around (Focus Group 4).

The participants made the following statements about being gaining independence:

I like workout myself, I just like working out with Adam (a classmate) (Focus Group 1).

…I could go to Lifetime and ask trainers to help me (Focus Group 3).

#### Happy

There were a total of 19 references made pertaining to participant Happiness. Within this theme, there were two subthemes identified as follows: Safety and Unhappy. The findings noted that the references made within this theme came from all four focus group data sources.

The participants made the following statements about becoming Happy:

Made more buddies. We play games and basketball. Buddies make me feel happy (Focus Group 1).

Buddies make me happy. Sarah and Hannah are my girls, they make me feel happy (Focus Group 1).

It feels great. I feel happy because it’s nice to be with friends here at [name] University. It’s a nice campus, my friends think so too (Focus Group 1).

The workout buddies think it is helpful for students to access or accommodate for needs on machines. Buddies help me and enjoy. Buddies feel pretty happy and seem excited to workout (Focus Group 2).

The participants made the following statements about becoming Safe:

More social skills, more confidence with machines, safety when working out (Focus Group 2).

Feel safe with buddy and willing to joke around (Focus Group 4).

There were two negative cases found as follow:

Think it is kinda boring for my buddy; enjoys talking with my buddy (Focus Group 3).

I think they enjoy, not sure how much they look forward to it (Focus Group 3).

#### Benefits

There were a total of 24 references made pertaining to participant Benefits. Within this theme, there were two subthemes identified as follows: Social and Workout. The findings noted that the references made within this theme came from all four focus group data sources.

The participants made the following statements about Social opportunities from engaging in the Workout/Recreation sessions:

We have a good time (Focus Group 1).

I am very talkative with my buddies now (Focus Group 1).

Spending time with buddies to gain friends and social skills. They like to know how our weekends and weekends were (Focus Group 2).

I am more outgoing because of buddies (Focus Group 2).

I have gained not to say “birthday” all the time. My social skills are better (Focus Group 2).

It seems fun, people look forward to seeing their buddies (Focus Group 2).

Gained …communication skills, friendship (Focus Group 3).

Feel a little more outgoing: feel more comfortable on campus (Focus Group 3).

Enjoy seeing what buddy likes and what I like are the same (Focus Group 4).

The participants made the following statements about Working out from engaging in the Workout/Recreation sessions:

Oh yeah, I like to workout with friends (Focus Group 1).

My friends help me workout (Focus Group 1).

The workout program is tons of help. Helps me to accomplish my goals (Focus Group 2).

Enjoy workout with buddies (Focus Group 2).

Gained strength (Focus Group 3).

Working out helps me to relax and not get stressed (Focus Group 4).

Buddies sometimes help muscles feel better (Focus Group 4).

When I feel tired, buddies help me feel better and make me work (Focus Group 4).

One negative instance was noted

Yes, not starting a conversation, but will talk if they start (Focus Group 4).

#### Assistance When Needed

There were a total of 14 references made pertaining to Assistance when Needed. Within this theme, there were two subthemes identified as follows: Help with Workout and Questions. The findings noted that the references made within this theme came from all four focus group data sources.

The participants made the following statements about obtaining Help.

They (buddies) help me (Focus Group 1).

My friends help me workout. We have a good time (Focus Group 1).

When I am lifting, I realize some weights can be changed…. More confidence with machines, safety when working out. I could go to Lifetime and ask trainers to help me (Focus Group 2).

Feel comfortable telling buddies if weights are too heavy, etc. (Focus Group 4).

They remind me to wear my heart rate monitor, they help me (Focus Group 2).

The participants made the following statement about asking Questions.

If I had any questions, they would answer them for me (Focus Group1).

#### Familiarity of Students and Environment

There were a total of 27 references made pertaining to participant Familiarity of Students and Environment. Within this theme, there were two subthemes identified as follows: Environment and Students. The findings noted that the references made within this theme came from all four focus group data sources.

The participants made the following statements about Familiarity with Students.

Sarah and Hannah are my girls, they make me feel happy (Focus Group 1).

Being around college peers is easy to make friends. Like spending time with workout buddies and spending time with them on campus. Easy to get along with. Easy for me to introduce myself (Focus Group 2).

When I meet new people I get really nervous and when I am with my buddies, I feel more comfortable (Focus Group 2).

People look forward to seeing their buddies (Focus Group 2).

The participants made the following statements about Familiarity with Environment.

It’s nice to be with friends here. It’s a nice campus, my friends think so too (Focus Group 1).

They enjoy working out with buddies. Like large recreation groups and games (Focus Group 2).

Feel more outgoing; feel more comfortable on campus (Focus Group 3).

#### Gaining Empowerment

From the data collected, one of the eight students gained empowerment in response between pre- and post-perceived leisure control scales pertaining to “getting people to do recreation activities even if they didn’t want to.” Barriers that the students were able to overcome due to this program include feeling comfortable around other college age peers, feeling comfortable when coming to a college campus to work/workout, having a feeling of being more outgoing due to the conversations with buddies, and gaining social skills that better their interactions with peers.

#### Assistance When Needed

Focus group data showed instances of the BOBW students feeling comfortable enough to ask for assistance when needed. Focus Groups 1 and 2 each showed an instance of being able to ask for assistance when they need it, “They remind me to wear my heart rate monitor, they help me” (Focus Group 1) and “If I had any questions, they would answer them for me” (Focus Group 2).

## Discussion and Recommendations

In general, the BOBW program had a positive impact on empowerment of BOBW students throughout the semester. All students reported that they enjoyed the program from the beginning of the semester to the end, giving the BOBW program a positive aspect in their life. This was similar to the findings of Green and Reese (21) who noted that by giving these students the opportunity to become empowered and be able to feel comfortable around others at the gym provides them with opportunities to obtain a healthy lifestyle. Also, all students reported feeling a great amount of help throughout the program from the beginning of the semester until the end. These positive instances showed an increase in empowerment on the students’ lives. Block et al. (9) noted similar findings that trying to eliminate barriers that were associated with disabilities and physical exercise help motivate and increase physical exercise in the students, as well as increasing support.

Similar to the findings of Block et al. (9), the program provided the students an opportunity of participation and involvement, the program was found to give them a sense of empowerment to make their own decisions and learn for themselves to become independent. Through participation with fitness and recreation programs, adults with disabilities learn to overcome self-imposed perceptions of their capabilities as well as how to turn their limitations into abilities. This was similar to Gabler-Halle et al. (16) who found that exercise has been shown to have a strong correlation between participation in an exercise program and positive changes in behavior for adults with disabilities. People who perceive themselves as competent, capable, and self-determining would be able to face and deal with life’s challenges (9). Findings of gaining empowerment were similar to those of Block et al. (9) in that giving students with disabilities the opportunity to participate in fitness and recreation programs, they are able to gain empowerment from the experience.

However, there were some factors that made the BOBW students feel less empowered by the program. The researcher noted during focus groups that some of the BOBW students were not confident in starting conversations with their university peer buddies. Although the BOBW students felt a sense of losing empowerment with this specific instance, there was an overall positive impact on the BOBW students’ empowerment. This is similar to what Tracy (19) noted, “Negative attitudes from peers can have a huge effect on the students and could potentially make the students feel uncomfortable and unwilling to come back” (19) (P. 347). By giving the students the opportunity to participate and socialize with peers their own age at a college setting, they were able to gain a sense of empowerment in their own life. The BOBW program university fitness center location was noted to be a friendly environment and this finding was contrary to that previously noted by Rimmer (5) who found the biggest barrier was that fitness and recreations facilities were unfriendly environments.

### Limitations to Study

Limitations to this study included the number of participants. Working with a limited group of students in the program made it difficult for the researcher to acquire many participants. Also, during focus groups, some participants had limited communication skills. For example, the researcher could only obtain one to three words or affirmative responses from two participants. Convenience scheduling of focus groups occurred due to the individualized BOBW student work and community schedules; therefore, groupings of the students may have reduced the depth of discussion or the content of the discussion. Also, this study was conducted over a very short time period. Additionally, one BOBW student had a scheduled operation and missed a couple of weeks of the program which could have impacted the results.

### Recommendations for Future Study

1. Given the fact that these research findings were based on data provided by eight BOBW Students over the course of a 3-month period, the first recommendation to be offered is that this study should be replicated on a broader scale with all BOBW students, as well as other transition programs that provide fitness and recreation opportunities.
2. Due to the limited empowerment theory literature within a transition program on a college campus, a longitudinal study should be employed to examine the empowerment of students by year of program as well as type and location of program.
3. Further study is also needed to determine if the impact of empowerment gained during the BOBW program impacts the students in other aspects of their lives as well as over the years.
4. Exploration is needed to determine how important empowerment is to gaining and maintaining jobs.

### Recommendations for BOBW Workout/Recreation Program

These results could suggest that in the future, training for both the BOBW students and their university buddies could center around how to best start conversations or find similar topics of interest for discussion. Part of the BOBW program outcomes are for the students to self-advocate, adding this information to the buddy training sessions could help identify when the BOBW students needs assistance versus when the college buddies are negatively impacting the empowerment of the BOBW students. Continuing the practice of providing additional socialization time outside of the workout and recreation environment may positively impact the previously noted barriers to empowerment.

## Author Contributions

ACS was the Primary Investigator of this study.

## Conflict of Interest Statement

The author declares that the research was conducted in the absence of any commercial or financial relationships that could be construed as a potential conflict of interest.
